# DKK-1 in prostate cancer diagnosis and follow up

**DOI:** 10.1186/1472-6890-14-11

**Published:** 2014-03-21

**Authors:** Patrizia D’Amelio, Ilaria Roato, Marco Oderda, Francesco Soria, Andrea Zitella, Riccardo Ferracini, Giulio Mengozzi, Paolo Gontero, Giovanni Carlo Isaia

**Affiliations:** 1 Gerontology Section, Department of Medical Sciences, University of Torino, Corso Bramante 88/90, 10126 Torino, Italy; 2 Center for Research in Experimental Medicine (CeRMS), Hospital City of Health and Science of Turin, Turin, Italy; 3 Urology Section, Department of Surgical Science, Hospital City of Health and Science of Turin, University of Turin, Turin, Italy; 4 Department of Orthopedics, Hospital City of Health and Science of Turin, Turin, Italy; 5 Baldi & Riberi Lab, Hospital City of Health and Science of Turin, Turin, Italy

**Keywords:** Prostate cancer, PSA, Dickoppf-1, DKK-1, Bone metastases

## Abstract

**Background:**

Dickoppf-1 (DKK-1) is a negative regulator of bone formation with tumorigenic potential. The up-regulation of DKK-1 is an early event in prostate cancer (PCa) development, thus we investigated its role as a marker in the diagnosis and prognosis of PCa.

**Methods:**

We retrospectively enrolled 159 patients who underwent prostate biopsy, either for elevated PSA or suspect digital rectal examination, between 2003 and 2010. During the biopsy, one serum sample was collected from all patients; PSA and DKK-1 were measured by ELISA technique. Amongst the biopsy of 159 patients 75 were affected by PCa and 84 were not the mean period of follow-up for these patients was 5 years; a new biopsy was performed in case of PCa suspicion.

**Results:**

PSA performed better than DKK-1 in detecting PCa (0.63 vs 0.51 respectively). Differently from PSA DKK-1 was significantly higher in patients who developed PCa during follow-up than in cancer-free ones, thus DKK-1 performed better than PSA in detecting these patients (0.67 vs 0.55). DKK-1 was significantly lower in patients with bone metastases, whereas PSA was not significantly different in patients with different outcomes.

**Conclusions:**

DKK-1 might be predictive for patients negative at first biopsy who will develop PCa and in the prognosis of bone metastases. It performed worse than PSA in the early diagnosis of Pca.

## Background

Prostate-specific antigen (PSA) is the most famous and debated cancer marker in the urological field. The recommendations of the European Association of Urology (EAU) Guidelines on Prostate Cancer (PCa) state that the main diagnostic tools for PCa include digital rectal examination (DRE), serum PSA level and trans-rectal ultrasounds (TRUS) [1]. Increasing levels of PSA are associated with an enhanced risk of the disease, but presently there is no upper or lower threshold limit [2].

PCa is currently the most frequently diagnosed cancer in males and constitutes a major health issue in developed countries, but the majority of PCa cases are considered clinically not significant and certainly not lethal. These discrepancies highlight the need for the early detection of PCa, especially for cases with aggressive features, which require an early and radical intervention. In these patients PSA is inadequate since it is prostate specific, but not a PCa specific marker: PSA increases in other common prostate diseases such as benign hyperplasia and prostatitis or after procedures as TRUS, biopsy and after transurethral prostatectomy. The use of PSA for predicting cancer aggressiveness and outcome is effective only for high PSA levels (>20 ng/mL) combined with Gleason score higher than 8 [3].

Continuous effort is made to find new reliable markers for the diagnosis and the prognosis of PCa, especially for PCa with low malignancy at histological evaluation. Among the potential markers, Dickoppf-1 (DKK-1) has shown interesting evidences, since its levels has been found elevated in different cancer types, such as multiple myeloma, gastric, lung, oesophageal and breast cancer [4-9] DKK-1 is a secreted inhibitor of the Wnt signalling pathway, which has tumorigenic and osteogenic potential [10-15]. Wnt proteins physiologically induce the differentiation and maturation of osteoblasts [16] and the secretion of Wnt proteins was shown to increase bone formation in osteoblastic metastases [17]. DKK-1 is a negative regulator of bone formation by antagonizing the Wnt pathway, and it is also involved in the proliferation of stem cells and tumorigenic processes [10-15].

The expression of DKK-1 in PCa samples is conflicting, because literature data report either an increase [18] or a non-significant change [19] in PCa samples. Recently an elegant study by Thudi et al. demonstrated a significant role for DKK-1 in PCa growth and ability to metastasise [12].

The present study aims to evaluate the potential usefulness of DKK-1 in diagnosis and prognosis of PCa patients with PSA levels lower than 20 ng/ml.

## Methods

This is a retrospective study, approved by the Ethical Committee of our Hospital (“Comitato Etico Interaziendale A.O.U. Città della Salute e della Scienza di Torino - A.O. Ordine Mauriziano - A.S.L. TO1”, that includes 159 men who underwent prostate biopsy due to suspicion of PCa, either for elevated PSA or suspect digital rectal examination, between 2003 and 2010. Patients consent to prostate biopsy, to collect and froze the serum was obtained at enrollment. Among this 159 men 75 were diagnosed with PCa and 84 were cancer free at biopsy. During biopsy a serum sample was collected and frozen at 80°C.We included in the study sera from patients with PSA lower than 20 ng/ml. DKK-1 levels were determined by a commercially available ELISA (BioMedica, Italy) in accordance with manufacturer instructions. The variability within- and between-run ranged from 6.5% to 8.0% and from 9.1% to 12.3%, respectively. PSA concentrations were assessed by electrochemiluminesce-based immunoassay automated on Cobas® analyzer (Roche Diagnostics GmbH, Mannheim, Germany).

**Figure 1:**
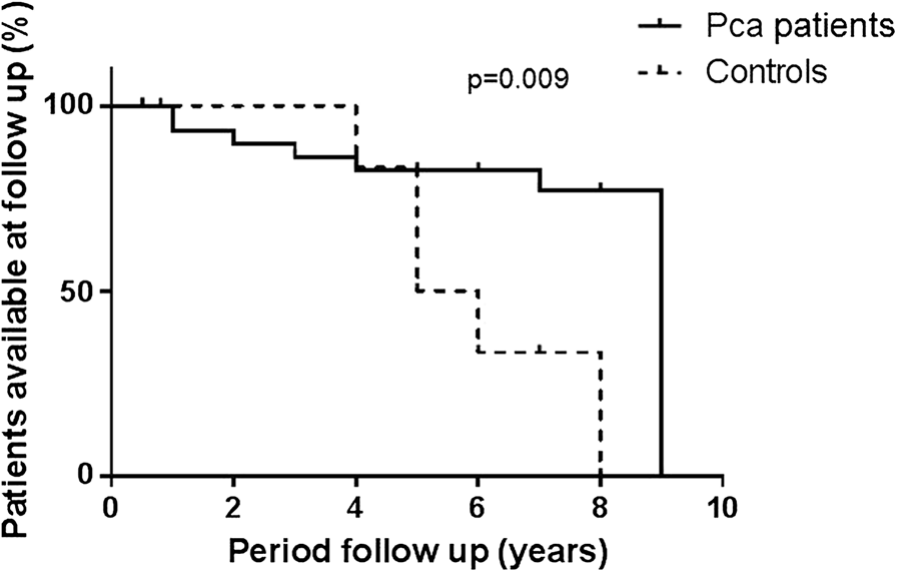
Survival curves show the availability of PCa patients (continuous line) and controls (dotted line) at follow-up the p value is showed.

The mean follow-up period for patients was 5 years (range 6 months-9 years); 28 subjects (17.6%) did not come back to follow-up, 12 of them died. The majority of subjects lost to follow-up were cancer free (Figure 1).

Follow-up consists of PSA measurement and DRE. A new biopsy was performed whether PSA rose: there was a suspect DRE or there was a previous diagnosis of atypical acinar proliferation. All PCa patients except for one, were subjected to radical treatment, either radical prostatectomy or radiotherapy.

During follow-up we identified 13 new PCa among subjects cancer free at enrolment.

In order to evaluate the prognostic role of DKK-1, patients were classified as: never had cancer, PCa-free, local recurrence, bone metastases, metastases other than bone at the last available follow- up. Among the 12 patients who died during follow-up only 4 died for cancer (6.8%).

### Statistical analyses

PCa patients and controls were compared for age: DKK-1 by means of one way ANOVA and PSA by means of Mann–Whitney test.

In order to evaluate prognostic value of DKK-1 as compared to PSA, patients and disease outcomes were compared for different values of PSA and DKK-1 after weighting cases for the follow-up period.

The Receiver Operating Characteristic (ROC) curves for PSA and DKK-1 were built and the area under ROC curves (AUROCs) compared both for diagnosis and prognosis.

SPSS 20.0 for windows software was used for statistical analyses; *p* values were considered significant when equal or lower than 0.05. Prism Graph Pad 6.02 for Windows was used to draw graphs.

## Results

### DKK-1 and PSA in PCa diagnosis

**Figure 2:**
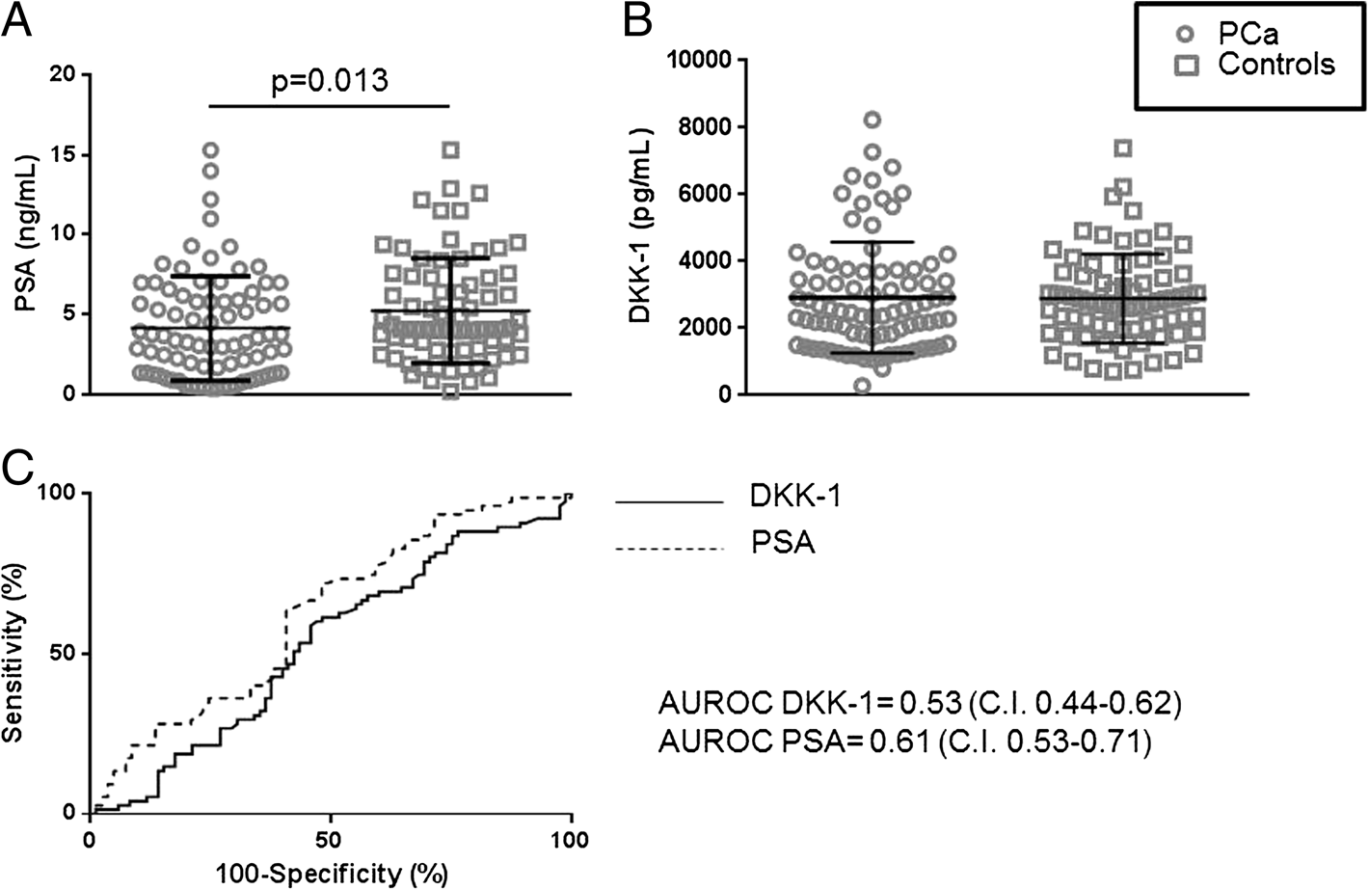
**Scatter plots show the level of PSA (panel A) and DKK-1 (panel B) in PCa patients and controls (significant p value is shown).** Panel **C** show the Receiver Operator Curves (ROC) for PSA (dotted line) and DKK-1 (continuous line), Area Under ROC (AUROCs) with CI are shown.

PCa patients (75) and controls (84) were comparable for age and DKK-1 serum levels, whereas PSA was significantly lower in non-cancer patients (Figure 2A, B). In order to evaluate whether PSA performed better than DKK-1 we carried out a ROC analysis, the PSA AUROC was slightly, although non-significantly, better that DKK-1 AUROC (Figure 2C).

The use of the ROC analyses allowed us to compare the diagnostic potential of PSA and DKK-1, the similar AUROC confirms that PSA lower than 20 ng/ml is not useful in the diagnosis of PCa.

### DKK-1 measured at diagnosis is lower in patients developing bone metastasis during follow-up

**Figure 3:**
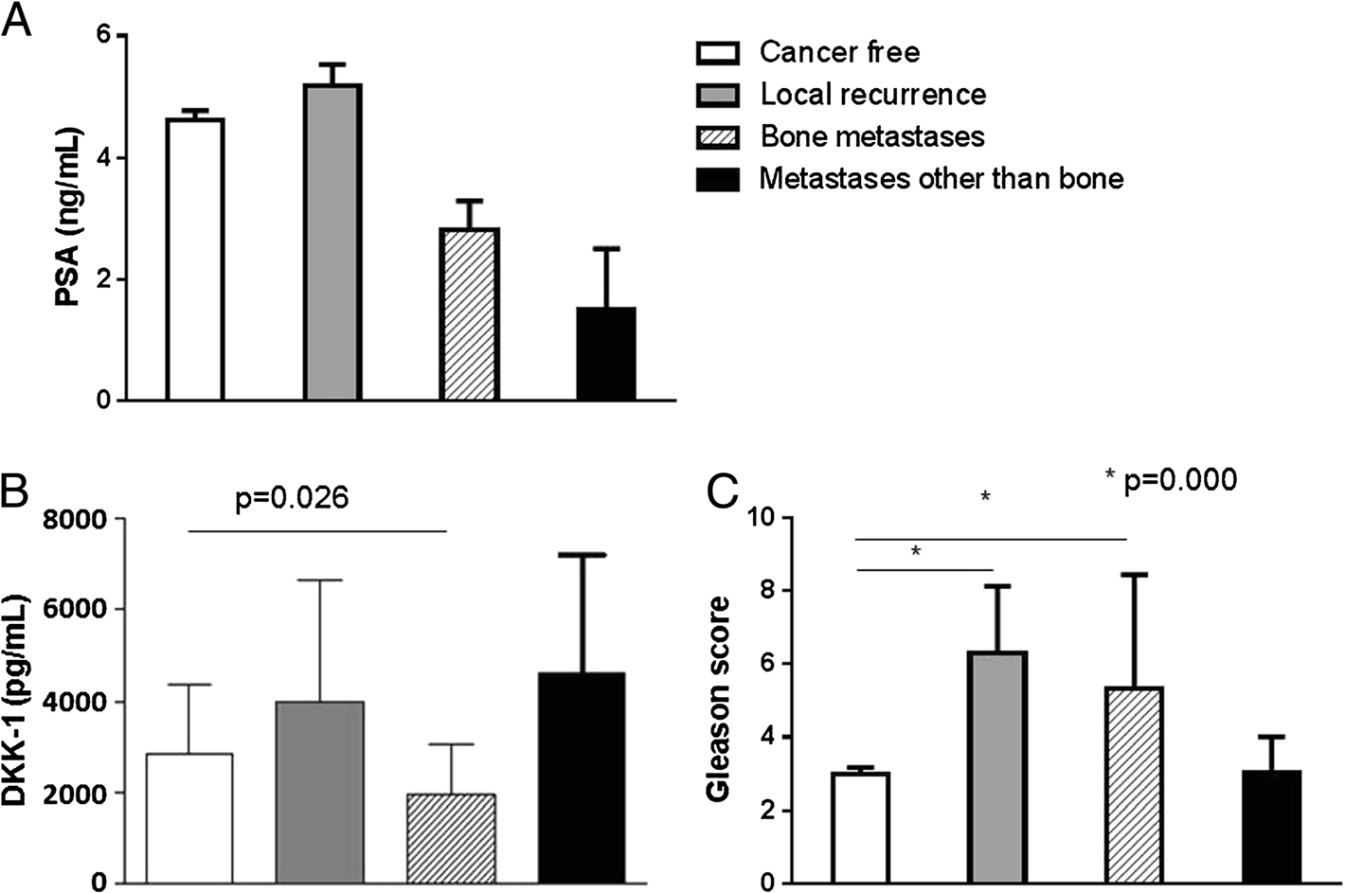
**Graphs show PSA (panel A), DKK-1 (panel B) and Gleason score (panel C) in relation with disease outcomes.** Bars represent mean and standard errors, p values were calculated with one way ANOVA for DKK-1 and Gleason; with Mann–Whitney test for PSA after weighting cases for period of follow-up.

At the last available follow-up, 75% of PCa patients were cancer free, 15.8% had local recurrence, 8.8% had osteoblastic bone metastases, 3.5% had metastases other than bone and 7% were dead for cancer related reasons. PCa patients’ outcomes were compared for PSA, Gleason score and DKK-1 after weighting cases for the duration of follow-up. PSA, measured at diagnosis, was not significantly different amongst different outcomes (Figure 3A), whereas DKK-1, measured at diagnosis, was reduced in patients that developed bone lesions during follow up (Figure 3B). Gleason score was higher in patients with local recurrence and in those developing bone metastases (Figure 3C).

### DKK-1 allow to identify new PCa at follow-up

**Figure 4:**
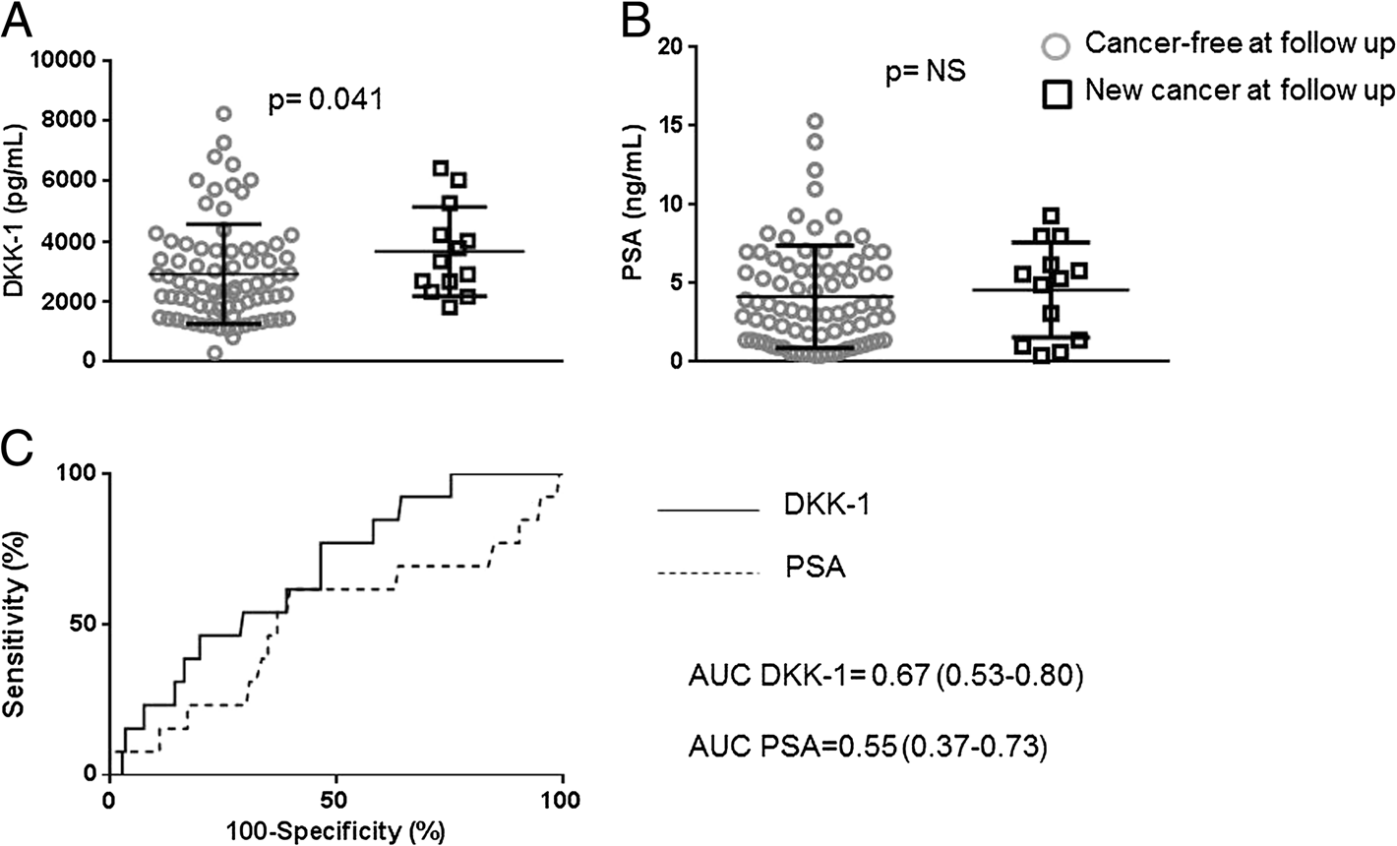
**Scatter plots show the level of DKK-1 (panel A) and PSA (panel B) in controls cancer- free at follow-up, and in patients with new diagnosed PCa (significant p value is shown).** Panel **C** shows the ROC curves for PSA (dotted line) and DKK-1 (continuous line), with AUROCs and CI.

Among subjects with negative bioptic findings at enrolment, 13 (15.5%) developed PCa. DKK-1 significantly increased in these patients, whereas PSA did not (Figure 4A,B). DKK-1 performed better than PSA, even though the AUROC was similar amongst the two markers (Figure 4C).

## Discussion

PCa is the most frequently diagnosed cancer in males, but very often it is not highly invasive. The wide adoption of PSA screening has been proved to increase diagnosis, to induce over treatment, to cause anxiety, treatment adverse events and to reduce the quality of life of patients [20,21]. For these reasons continuous efforts are made to identify new more reliable markers for the diagnosis and prognosis of PCa other than PSA.

It is well known that aggressive PCa very often causes bone metastases [22], which are typically characterized by excessive bone formation: highly active osteoblasts form structurally weak and sclerotic bones at high risk of fracture. DKK-1 is a fundamental inhibitor of the Wnt pathway [10], controlling the formation and activity of osteoblasts, and it is impaired in osteoblastic bone metastases [7,23].

Recently DKK-1, as a molecule involved in the control of bone formation, has shown to be involved in PCa biology and in its propensity to develop bone metastases [12]. We previously showed that DKK-1 was elevated in sera of PCa patients [18]. DKK-1 was directly produced by PCa cells, whereas normal prostate tissue did not produce this molecule [18]. In the present study, DKK-1 was not significantly higher in PCa patients than in controls, appearing discordant by the above reported data, nevertheless this may be explained by the different origin of the control group. Indeed, now we compared PCa patients to subjects showing an increased PSA with a benign prostatic disease, whereas in the previous study the control group was constituted by healthy subjects [18]. Here we show that DKK-1 is decreased in PCa patients who will develop bone metastases, whereas PSA is not significantly different in these set of patients. These data are consistent with the role of DKK-1 in the control of cancer progression and of bone formation. A recent study by Thudi et al. demonstrated that in PCa over expressing DKK-1 there was an increased in the growth and metastatic activity of PCa cells but a decreased bone formation in bone metastases [12]. These results confirmed previous data showing that DKK-1 expression is an early event in PCa. During PCa progression DKK-1 expression decreases, particularly in advanced bone metastases [24]. These data support a model in which DKK-1 acts as a molecular switch promoting the transition of bone lesions from osteolytic to osteoblastic [24]. The biological functions of DKK-1 in the bone metastatic process and in tumor progression suggest its potential therapeutic role as a target [25].

DKK-1 did not add significant information to PSA in the early detection of PCa, although the AUROC for DKK-1 and PSA was comparable. This result should be considered despite several limitations of the study, such as the retrospective design, the small number of patients enrolled and the short follow-up, considering the long natural history of PCa. Noteworthy, we showed that DKK-1 was more elevated in patients with negative bioptic finding further developed PCa during follow-up, whereas PSA was not significantly different in these subjects at enrollement. The potential ability of DKK-1 to detect PCa patients with higher risk of progression would add valuable information to the risk stratification in clinical practice. This is particular important in order to increase the ability to predict PCa development; although only 13 patients developed a new cancer during follow up, the ROC analyses showed that DKK-1 performed slightly better than PSA in detecting these patients. Our results confirmed literature data reporting the role of DKK-1 as a potential serological biomarker in different tumors such as gastric cancer [26], hepatocellular carcinoma [27,28], non-small cell lung cancer [29] and gynaecological cancer [5].

Our findings need to be confirmed in a larger cohort of patients. DKK-1 could be useful in the difficult management of patients with suspicion of harbouring PCa despite a previously negative biopsy. Such patients are a challenge for the urologists: after an initial negative bioptic finding, a further biopsy has shown to be positive in 10-35% of cases [30].

## Conclusion

In summary, our study unravelled a potential utility of DKK-1 in the diagnostic process of PCa. Indeed, in our cohort of patients, DKK-1 detects patients with high risk of PCa progression and a previous negative biopsy. Further studies on larger scale are surely warranted, keeping in mind that this marker could also represent an interesting therapeutic target to disrupt the metastatic process of PCa.

## Abbreviations

DKK-1: Dickoppf-1; PCa: Prostate cancer; PSA: Prostate-specific antigen; DRE: Digital rectal examination.

## Competing interests

The authors declare that they have no competing interests.

## Authors’ contributions

PD conceived of the study, and participated in its design and coordination, performed the statistical analysis and helped to draft the manuscript. IR participated in its design and coordination and helped to draft the manuscript. MO, FS, AZ, PG collected the samples, collected the data and helped to draft the manuscript. RF and GCI participated in the coordination of the study and helped to draft the manuscript. GM performed the ELISA assays and helped to draft the manuscript. All authors read and approved the final manuscript.

## Authors’ information

PD, RF and CGI are MDs interested in bone metabolism diseases and published several translational research works, these authors are mainly interested in diseases pathophysiology.

IR is a PhD, she works on the mechanism of bone metastases formation.

MO, FS, AZ and PG are urologist mainly interested in prostatic disease.

GM is a doctor mainly interested in the study and development of new biomarkers.

## Pre-publication history

The pre-publication history for this paper can be accessed here:

http://www.biomedcentral.com/1472-6890/14/11/prepub
